# Effect of preparation conditions on the properties of nano ZnO powders during ultrasonic assisted direct precipitation process

**DOI:** 10.1371/journal.pone.0286765

**Published:** 2023-08-31

**Authors:** Jingfeng Wang, Haiyang Ji

**Affiliations:** Zhengzhou Railway Vocational and Technical College, Zhengzhou, Henan, PR China; Nazarbayev University, KAZAKHSTAN

## Abstract

Transparent conductive thin films (TCO) are widely used for their excellent photoelectric properties. To prepare high-quality ZnO targets, starting with the original ZnO powder is necessary. This paper aims to explore the basic technology and method of ultrasonic-assisted direct precipitation for mass production of ZnO powder and to analyze the effects of factors such as precipitating agent, surfactant, calcination temperature, and solvent on the powder’s morphology, particle size, and crystallinity. The study found that the type and amount of precipitants and surfactants affect the powder’s morphology and dispersibility, while calcination temperature mainly affects the powder’s morphology and crystallinity. The ethanol content in the solvent mainly affects the grain size. After testing different variables, the optimal conditions for preparing spherical ZnO powder were found to be using (NH_4_)_2_·CO_3_ as the precipitant, adding 3% wt of PEG-400 and 3% wt of TEA at a calcination temperature of 320°C and a 60% ethanol solvent. This resulted in a smooth surface, uniform particle size distribution, good dispersibility, high crystallinity, and particle sizes between 26-32nm.

## Introduction

Currently, the main applications for TCOs are indium tin oxide (ITO) and fluorine-doped tin oxide (FTO), which have both advantages and disadvantages. The source materials for producing ITO targets are expensive, especially the rare metal indium, and the equipment required for preparing them is also relatively costly, resulting in a higher price for ITO targets [1–3]. Additionally, ITO thin films are vulnerable to environmental influences, particularly temperature, which weakens their light transmittance at high temperatures [4]. Therefore, current research has partially shifted to ZnO thin films [5–8]. The raw materials for preparing ZnO thin films are abundant and cost-effective. ZnO thin films have good transmittance and conductivity properties (85% and 10^−4^ Ω·cm, respectively) [9]. Moreover, the performance of ZnO thin films is relatively stable during use, and they do not react in hydrogen ion environments like ITO. Doping specific elements can further enhance the performance of ZnO thin films, making them a potential substitute for ITO thin film materials and a new type of TCO thin film material [9–11].

To prepare high-quality ZnO thin films, it is necessary to use ZnO targets with high density and conductivity. Many factors affect the relative density of ceramic targets during their preparation, with the most important being the sintering process and the original powder [12–14]. Apart from selecting the best sintering process, an appropriate initial powder should also be chosen. In general, spherical fine particle powders have a large sintering driving force during the sintering process, and the atomic diffusion distance is shortened, which accelerates the densification process of the target material. M. Sabzi et al. [15] studied the influence of different sintering parameters on the microstructure evolution, density, and hardness of nano powder WC parts and found that the best strength of WC is achieved at 1400°C, mainly because the surface energy increases with decreasing particle size. Therefore, nanoscale powders smaller than 100 nm provide high driving force for sintering and allow sintering to be carried out at lower temperatures. Moreover, spherical powders have the lowest surface volume ratio and therefore have the lowest surface energy compared to other forms. Chen et al. [16] studied the sintering characteristics of CeO_2_ and Y_2_O_3_ powders and found that very fine, surface-active powders with good sintering properties can be easily sintered to full density. Fang et al. [17] used experimental data on sintered nano tungsten carbide and tungsten powder and selected data from other materials in the literature to study the unique characteristics of nano sintering. They found that narrow particle size distribution and high green density are conducive to achieving maximum densification with minimal grain growth. If the original particle size and shape are not uniform, secondary recrystallization may occur during the sintering process. If there are a small number of large particles, it is extremely unfavorable for sintering, which not only affects the microstructure of the material but also affects its macroscopic properties. In short, while ensuring the high purity of the powder, the monomerization of the raw material powder and the homogenization of the composition and organizational structure are new directions for the preparation of target materials.

There are many methods for preparing ZnO powder, which can be divided into physical and chemical methods [18–21]. The advantage of physical methods lies in their simplicity and ease of operation, which allows for large-scale production of ZnO powder compared to other methods. However, it is difficult to control the particle size, shape, and distribution of ZnO powder prepared by physical methods. In contrast, the chemical method consumes less energy and is more economical. Additionally, the particle size and shape of ZnO powder prepared by the chemical method can be controlled by adjusting the production process. However, in the mass production of powders, the reaction process is complex and difficult to control [21]. Huang et al. [22] prepared large-scale flower-shaped ZnO nanostructures using a very simple solution method at room temperature. The flower-shaped ZnO nanostructure is self-assembled from thin and uniform nanosheets with a thickness of approximately 18 nm. Narges Kiomarsipour et al. [23] synthesized well-dispersed hexagonal-structured ZnO using zinc nitrate hexahydrate as raw material and a low-temperature hydrothermal process. ZnO nanorods with diameters of about 50, 200, and 500 nm and lengths of 300 nm, 1 μm, and 2 μm were formed on a large scale using different temperatures. Ma et al. [24] successfully synthesized a large-scale, uniform mulberry-like ZnO powder using a fast and simple microwave-assisted hydrothermal method, with an average diameter of approximately 150 nm.

While some studies have attempted to produce ZnO powders on a large scale, the resulting nano ZnO powders are not suitable for sintering ZnO targets due to their inappropriate shape and size. In this paper, we employed the ultrasonic-assisted direct precipitation method to prepare a large quantity of ZnO nanopowder, and investigated the influence of factors such as the precipitant, dispersant, calcination temperature, solvent, and others on the morphology, particle size, and crystallinity of ZnO.

## Materials and methods

### Materials

All reagents used in this study were of analytical purity, including Zn(NO_3_)_2_·6H_2_O、NH_3_·H_2_O、Na(OH)、TEA、PEG-400、PVP-K30、PVA100-40H, were purchased from Tianjin Kemio Chemical Reagent Co., Ltd.

### Sample preparation

The main experimental process steps, using Zn(NO_3_)_2_·6H_2_O and (NH_4_)_3_CO_2_ as examples, are as follows: Zn(NO_3_)_2_·6H_2_O and (NH_4_)_3_CO_2_ were firstly dissolved in high-purity water to form solutions with 1 mol/L and 1.5 mol/L concentration, respectively. The Zn(NO_3_)_2_ solutions were slowly dropped into the (NH_4_)_2_CO_3_ solutions with simultaneously performing ultrasonic treatment until the pH value of the solution reaches 7.0. The pH value of the solution is measured and adjusted with HNO_3_. The stable solution is kept at a constant temperature of 80°C for 2 hours. The sediment and solvent are separated by suction filtration and continuously washed with ethanol. After multiple washings, the cleaned precursor is dissolved in ethanol again and subjected to ultrasonic treatment for 15 minutes. The washed precipitate is then dried in an electric heating oven at 80°C for 24 hours to obtain ZnO precursor. After sufficient drying, the precursor is placed in a corundum crucible and calcined in a resistance furnace at a temperature and time set according to the experimental purpose to obtain ZnO powder.

### Characterization

In this paper, the ZnO powder prepared under different conditions was characterized using the XRD 6000 type X-ray diffractometer from Shimadzu Corporation of Japan, in order to analyze the phase composition of the prepared powder. The morphology, particle size, and dispersion of the ZnO powder were observed using a Philips Quanta-2000 scanning electron microscope. The particle size and distribution were automatically obtained by performing simple grain count statistics using Nano Measure 1.2 software.

## Results and discussion

### Effect of different precipitators on the properties of ZnO powder

**Fig 1:**
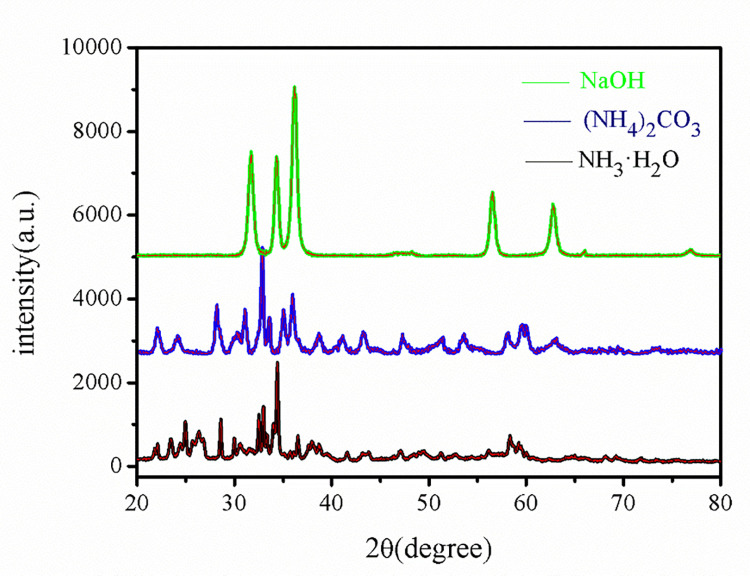
Precursors prepared with different precipitators.

In this paper, NH_3_·H_2_O, (NH_4_)_2_·CO_3_, and NaOH were used as precipitants to prepare precursors. NH_3_·H_2_O is a weakly alkaline solvent with strong polarity, (NH_4_)_2_·CO_3_ is easily soluble in water, and its aqueous solution is alkaline. During the reaction process, it decomposes into CO_2_, which acts as a micromixer and increases the distance between particles. NaOH is a strong caustic alkali that generates a large amount of heat in a short time after being dissolved in water. Fig 1 shows the XRD pattern of the precursors obtained using these three precipitants.

**Fig 2:**
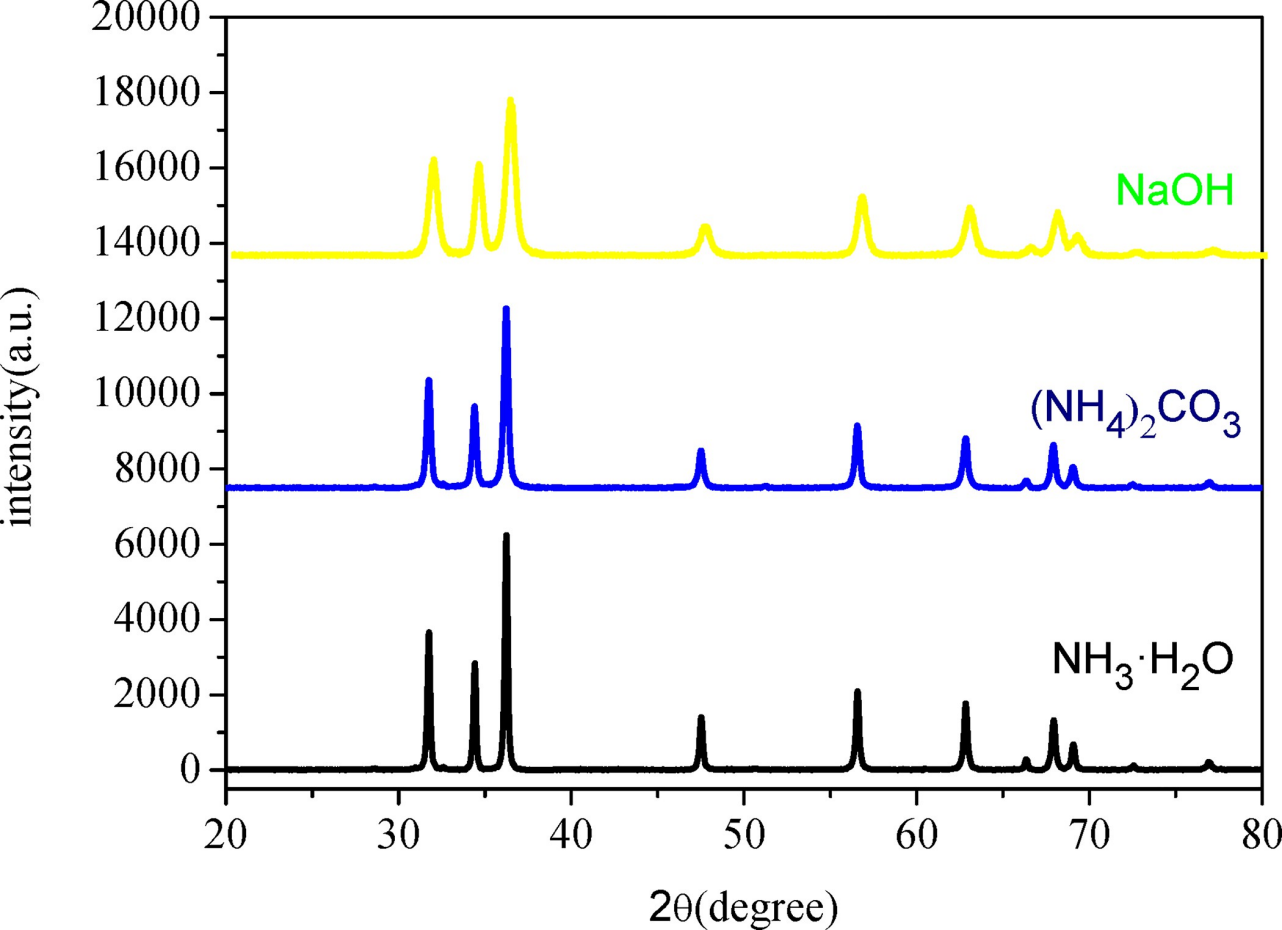
XRD pattern of powder after precursor calcination.

It can be observed that the powders obtained by calcining the three precursors are all ZnO, as shown in Fig 2. Based on the analysis of the obtained spectra, the ZnO spectra obtained after calcination of the three precursors are only different in strength, which is consistent with the standard card JCPDS36-1451 [25].The crystal structure is hexagonal wurtzite, with lattice structure parameters a = 3.24982 and c = 5.20661, belonging to the P63mc spatial point group. The prepared ZnO powder exhibits seven obvious diffraction peaks, and the positions and relative intensities of these diffraction peaks are consistent with the standard card of ZnO. The diffraction angles corresponding to the diffraction peaks are 2θ = 31.69°, 34.36°, 36.18°, 47.54°, 56.51°, 62.78°, 66.30°, 67.96°, and 69.10°. These diffraction angles correspond to (100), (002), (101), (102), (110), (103), (200), (112), and (201) crystal planes, respectively [26, 27].

**Fig 3:**
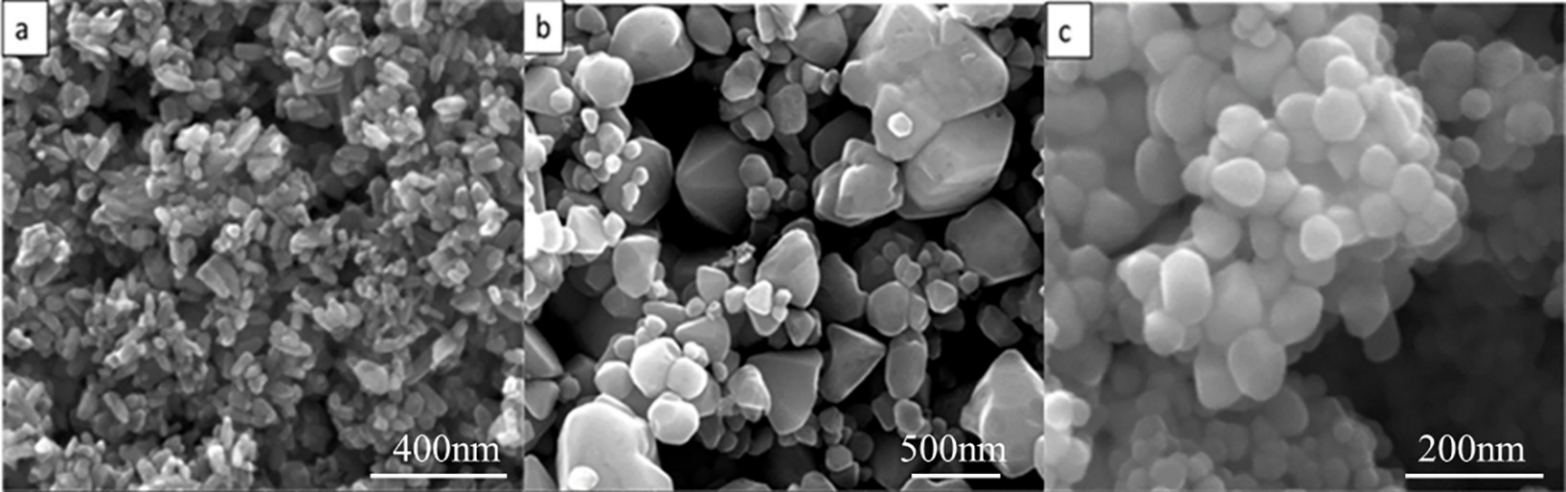
Morphology of different precursors after calcination. (a) NaOH precipitator; (b) NH_3_·H_2_O precipitator; (c)(NH_4_)_2_·CO_3_ precipitant.

To observe the prepared ZnO powder, SEM was used. Fig 3 displays the morphology of the three types of ZnO powder. As seen from Fig 3, the morphology of the ZnO powder prepared using NaOH as a precipitant is short rod-shaped, and agglomeration is apparent, forming flower-shaped blocks. This is because NaOH is a strong alkali, and the intensity of ultrasound and stirring is limited, which is not sufficient to quickly and evenly disperse the added NaOH, resulting in local excessive alkalinity [28, 29]. At the same time, a large amount of exothermic reaction leads to powder agglomeration. Additionally, the addition of NaOH results in polar growth of the powder, with the grain rapidly growing in the [1] direction and eventually growing into a rod shape [30]. The ZnO powder obtained by using NH_3_·H_2_O as a precipitant has good dispersion, but its particle morphology is extremely irregular, and its particle size distribution is wide. The ZnO powder obtained by using (NH_4_)_2_·CO_3_ as a precipitant has a regular particle shape, narrow particle size distribution, and a particle diameter of about 50 nm, but significant agglomeration. The continuous decomposition of (NH_4_)_2_·CO_3_ in water makes it easier to maintain a relatively stable solution concentration, which can promote the orderly growth of powders [18]. Moreover, experiments have shown that the droplet acceleration of the precipitant has a significant impact on the final particle size of the powder. If it is too fast, it will lead to larger particle sizes and severely uneven particle size distribution. If it is too slow, the reaction cycle will be longer, which is detrimental to production efficiency.

### Effect of surfactant type and content on powder properties

Different surfactants have various dispersion mechanisms, including electrostatic stability, spatial steric resistance, and space potential stabilization mechanisms [31]. The electrostatic mechanism involves adjusting the solution’s acidity or adding an electrolyte to attract hetero-electric ions on the particle surface, forming a double electric layer. The Coulomb force between the double electric layers increases, reducing the gravitational force between particles and causing them to disperse. In the spatial steric resistance mechanism, particles are dispersed in organic solvents with low electrical constants, resulting in physical isolation. The space potential stabilization mechanism combines both space steric resistance and electrostatic repulsion. The nanoparticles’ ability to adsorb electrolytes is related to the solution’s pH value. Adjusting the solution’s pH value can saturate and maximize the ionization of the electrolyte adsorbed on the nanoparticles’ surface, thereby increasing the electric double layer’s repulsion force and achieving even and stable particle dispersion. In this study, we used (NH_4_)_2_·CO_3_ as a precipitant to investigate the effects of different types and amounts of surfactants on powder quality.

**Fig 4:**
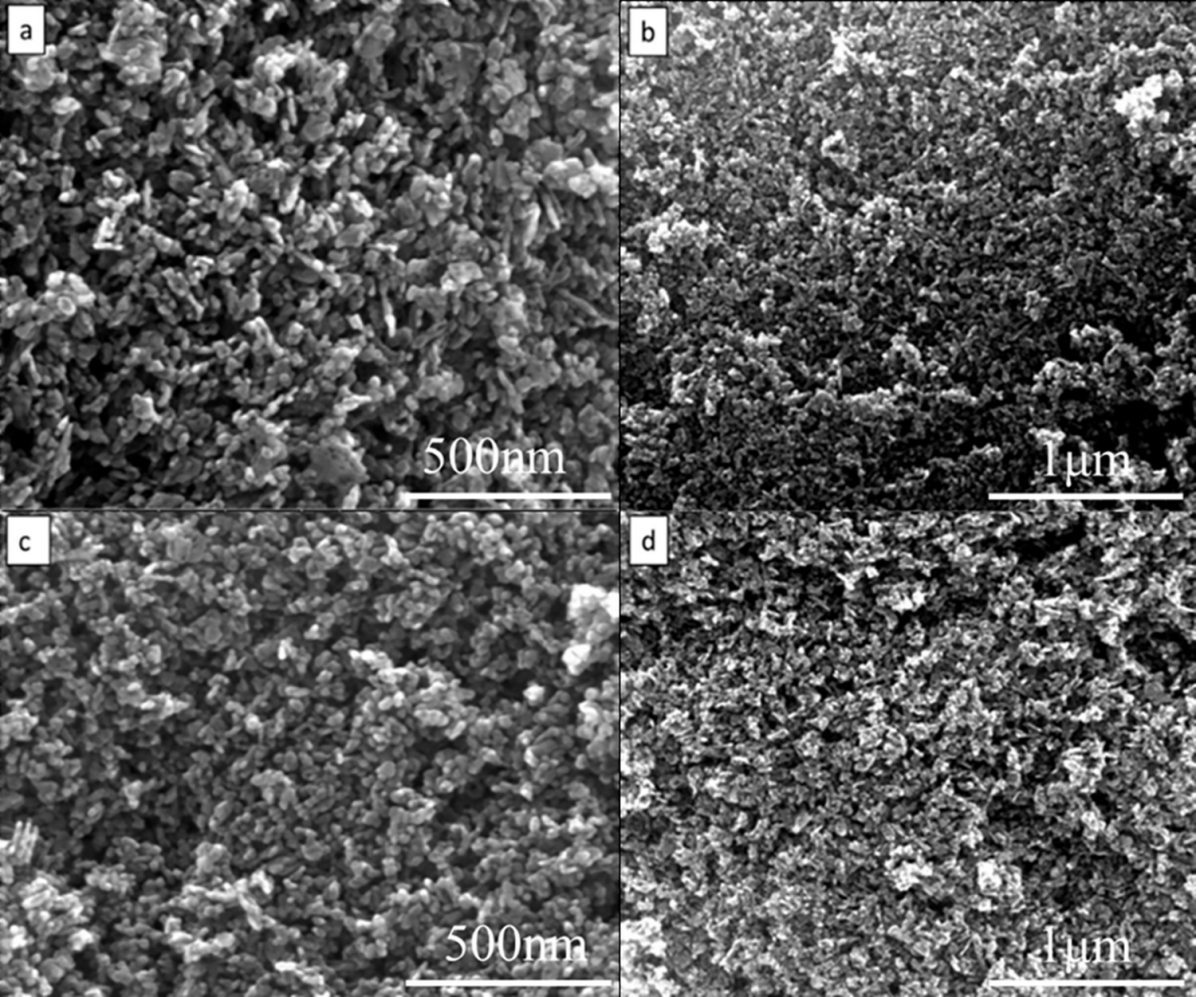
Morphology of ZnO powders prepared with different contents of PVP-k30. (a) 0.5%; (b) 1%; (c) 3%; (d) 5%.

Fig 4 shows a scanning electron microscopy (SEM) image of ZnO powder prepared using varying amounts of PVP-K30 surfactant. Since PVP-K30 is a nonionic polymer compound, its dispersion mechanism primarily involves reducing the effect of empty spaces on the dispersion of ZnO powder [32]. This results in changes to the particle morphology, making the grain size of ZnO more uniform and leading to a refinement of the ZnO powder. It can be observed that the addition of PVP-K30 has a minimal impact on the morphology of ZnO powder, but results in increased aggregation. This phenomenon occurs due to the adsorption of PVP-K30 on the charged solid surface caused by its polarity. In terms of the size of ZnO particles, the addition of 3% PVP-K30 leads to the smallest particle size and a spherical shape. This effect could be attributed to the adsorption of the polar surface of PVP on the atomic nucleus, which interferes with the equilibrium and reduces the growth rate along the c-axis. Therefore, growth along the non-polar surface is preferred, leading to the gradual formation of the final spherical shape. M. Bitenc et al. [32] utilized a straightforward method involving urea aqueous solution and zinc nitrate as the starting salt to synthesize zinc hydroxide carbonate and ZnO microcrystals. They also added a small amount of different additives, such as PVP-K30, during the synthesis process, and the results demonstrated that this altered the morphological characteristics of zinc-based precipitates. Alan Meng et al. [33] successfully prepared ZnO/Ag nanocomposites composed of quasi-spherical nanoparticles with a diameter of several nanometers using a two-step liquid-phase precipitation method. They proposed that non-ionic dispersants can form adsorbed PVP molecular layers on the surface of ZnO/Ag nanoparticles, preventing their agglomeration and improving their dispersion stability in isopropanol.

**Fig 5:**
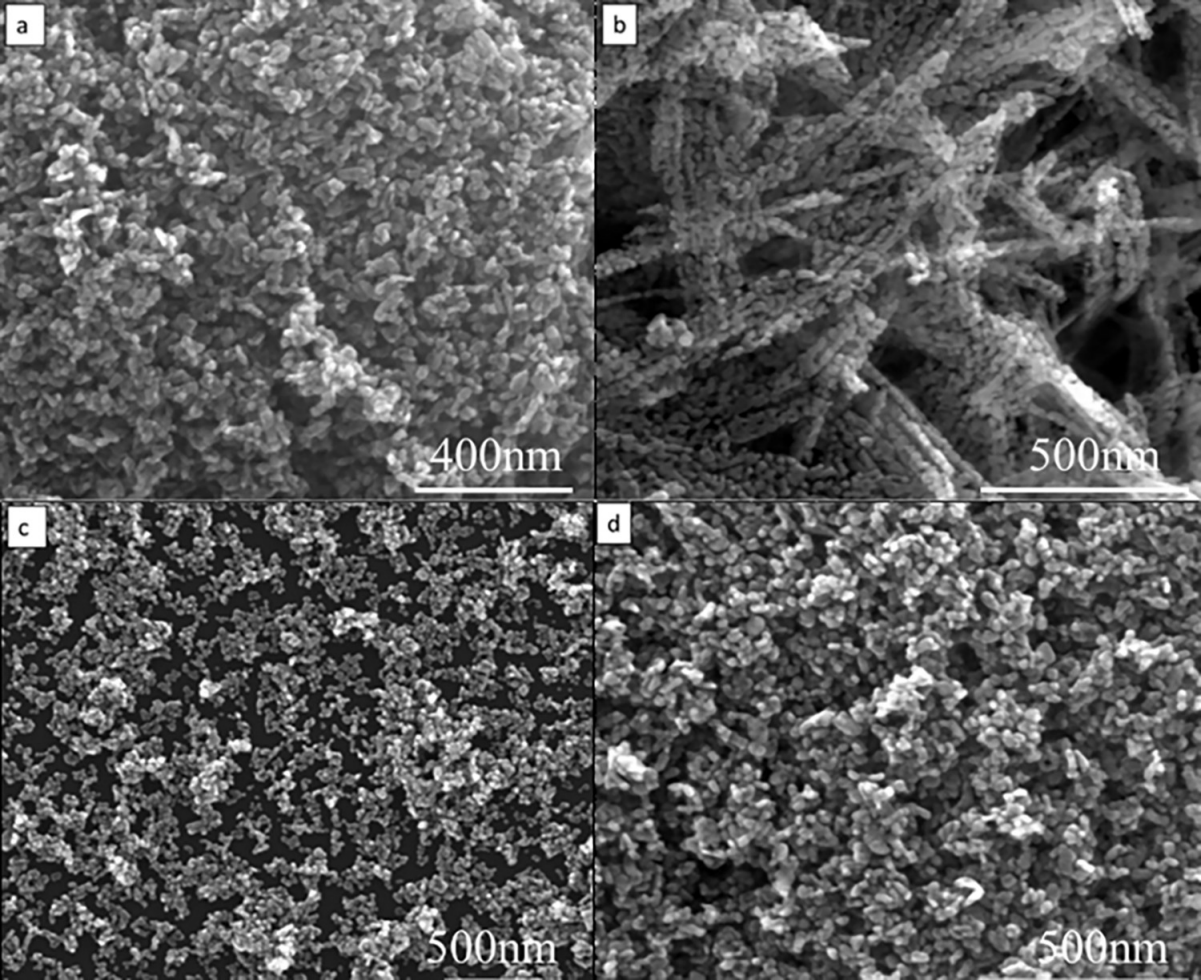
Morphology of ZnO powder with different PEG-400 additions. (a) 0.5%; (b) 1%; (c) 3%; (d) 5%.

PEG-400 is a long-chain macromolecule that readily adsorbs onto the surface of metal oxide colloids. When the polyethylene glycol macromolecules adsorb onto the powder’s surface, they significantly reduce its surface energy, slowing the growth rate of the colloids in a particular direction and leading to anisotropic crystal growth [34, 35]. Moreover, PEG-400 effectively promotes the nucleation of ZnO and impacts its growth process. As shown in Fig 5(A), at an addition amount of 0.5%, agglomeration is still quite evident. When the addition amount is 1%, as in Fig 5(B), the particles are significantly dispersed, but a network distribution of particles forms, possibly due to the addition of PEG-400 as a soft template. At 3%, as seen in Fig 5(C), the agglomeration reduces. Conversely, as illustrated in Fig 5(D), at an addition amount of 5%, the powder again becomes agglomerated. This may result from the excessive amount of additives, which increases the solution viscosity, causing the nanopowders to stick together and agglomerate.

Triethylamine (TEA) is slightly soluble in water but soluble in ethanol and ether. Its aqueous solution is alkaline, which can cause particles to tend to be spherical. Adding TEA to the solution can effectively modify the particle surface, reduce the particle surface energy, and promote the growth of ZnO powder in a spherical shape. A. K. Singh et al. [36] synthesized nano zinc oxide using chemical methods in alcohol-based solvents. TEA was used as a capping agent during the synthesis process to synthesize spherical ZnO particles with particle sizes ranging from 40 to 50 nm. In another study by Amir Kajbafvala et al. [37], spherical ZnO nanoparticles with a diameter of 600 nm were prepared using butanol, triethanolamine (TEA), and zinc acetate dihydrate as raw materials.

**Fig 6:**
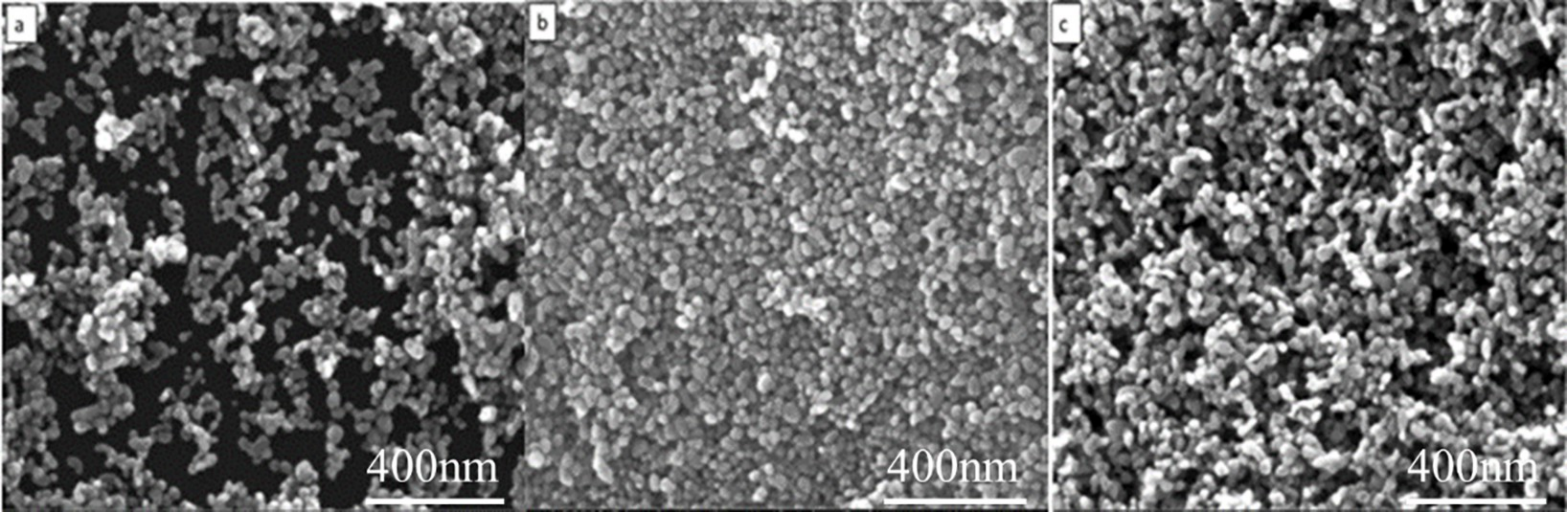
Morphology of ZnO powder with different TEA additions. (a) 1%; (b) 3%; (c) 5%.

Additionally, TEA can be adsorbed on the surface of ZnO powder to a certain extent, reducing the electrostatic attraction of nanoparticles and improving their dispersion. Fig 6 shows the nano ZnO powder prepared by adding different mass fractions of triethylamine (TEA) to a PEG-400 solution with a mass fraction of 3%. As seen in Fig 6(A), adding 1% wt of TEA leads to the particles growing in a spherical shape, with some local agglomerations. Furthermore, statistical analysis of particle size distribution using Nano Measure software showed that the diameter of the powder decreased to about 34 nm, with a uniform particle size distribution. This indicates that the addition of TEA effectively modifies the surface of nanoparticles, affecting the growth process of ZnO powder, and promoting their spherical shape. As seen in Fig 6(B), when 3% TEA is added, the morphology of ZnO powder is spherical, with a smooth surface, uniform particle size distribution, and good dispersion. The grain size is mostly between 26 and 32 nm, with a relatively uniform particle size distribution. From Fig 6(C), it can be observed that when 5% TEA is added, the particle size of ZnO powder does not change much, but each particle is connected end-to-end, with a tendency to form rod shapes. With regard to the effect of the additive on morphology, an adsorption mechanism seems to be most reasonable. In reaction system, it is suggested that TEA adsorbs to some extent on the basal crystal surface of ZnO and thus inhibits the growth along the c axis; the preferred growth direction. As a result, growth is equally favored in all directions resulting in spherical morphology which agglomerate [36].

It can be concluded from Figs 4(C), 5(C), and 6(V) that the addition of different additives produces varying morphologies of ZnO powder. This is due to the diverse effects of different additives on the powder, as well as their varying dispersion mechanisms. The dispersion effect on ZnO powder is dependent on the type and amount of additive added. As shown in Fig 4, PVP-K30, a nonionic polymer compound, has a poor dispersion effect on nano ZnO powder, as agglomeration is still significant after addition. However, the grain morphology is relatively regular. When PEG-400 is used as a dispersant and added to the solution, it adsorbs on the surface of the metal oxide colloid, reducing its surface energy greatly. The dispersion mechanism is primarily the vacancy barrier effect, which separates the grains and weakens the particle growth rate in a particular direction, resulting in anisotropic growth and ultimately obtaining ZnO powder with a more refined, regular shape and good dispersion, as shown in Fig 5.

Fig 6 illustrates that the addition of TEA effectively adjusts the nucleation rate and increases the crystal growth rate, resulting in spherical ZnO nanoparticles with excellent dispersion. Spherical nano ZnO powders have a relatively large surface area, and the larger contact area between particles, compared to dendritic and needle-shaped powders, makes it easier to form high-density targets during burning.

### Effect of calcination temperature on powder properties

The precursor prepared through the direct precipitation method requires calcination before it can be decomposed into ZnO powder, so it is crucial to select an appropriate calcination temperature. Different calcination temperatures can significantly affect the properties of ZnO powder [38, 39]. A temperature that is too high can result in grain coarsening, while a temperature that is too low can decrease the crystallinity of ZnO.

**Fig 7:**
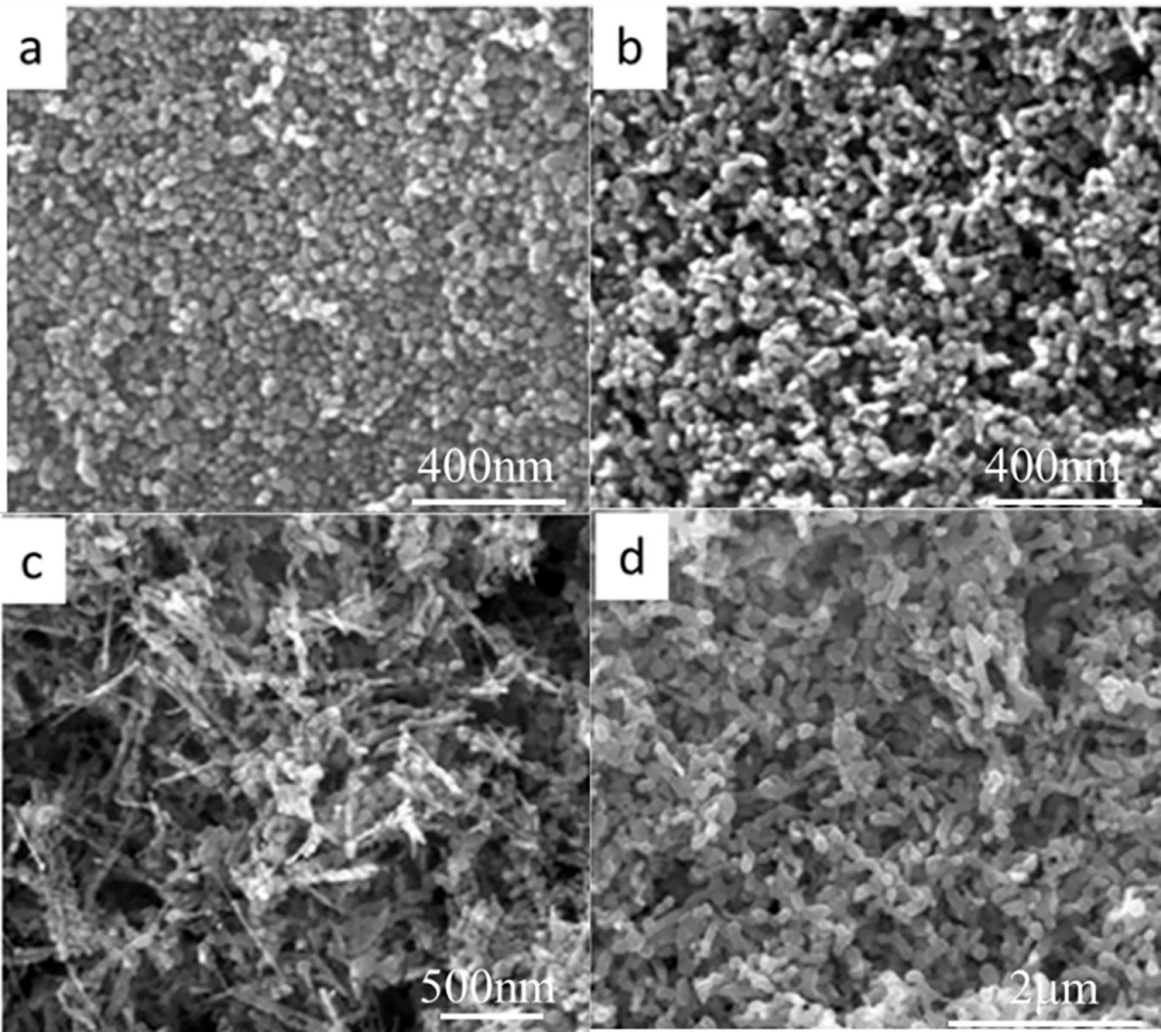
Morphology of ZnO powder at different calcination temperatures. (a) 320°C;(b) 400°C; (c) 500°C;(d) 600°C.

The precursor obtained through the direct precipitation method needs to undergo calcination to decompose into ZnO powder, and choosing an appropriate calcination temperature is crucial. Different calcination temperatures can significantly affect the properties of ZnO powder. If the temperature is too high, it can lead to grain coarsening, while if it is too low, it can lower the crystallinity of ZnO. In this study, we investigated the effect of calcination temperatures of 320°C, 400°C, 500°C, and 600°C. As shown in Fig 7, the X-ray diffraction pattern of ZnO powder obtained at different calcination temperatures revealed the same crystal form without any impurity peaks. With increasing calcination temperature, the diffraction peak became sharper, the intensity increased, and the half peak width narrowed. These results indicate that the particle size increased, and the crystal form tended to be complete at higher temperatures. Zhou et al. [40] and Naif Mohammed Al-Hada et al. [41] have also reported a similar phenomenon in which the average particle size and crystallinity increase with increasing calcination temperature. These findings suggest that as the temperature rises, adjacent particles tend to fuse together by melting their surfaces, resulting in larger particle sizes. Fang et al. [42] propose that at high temperatures, atoms are given more energy to diffuse and settle in their appropriate positions in the lattice, and grains with lower surface energy tend to grow larger.

**Fig 8:**
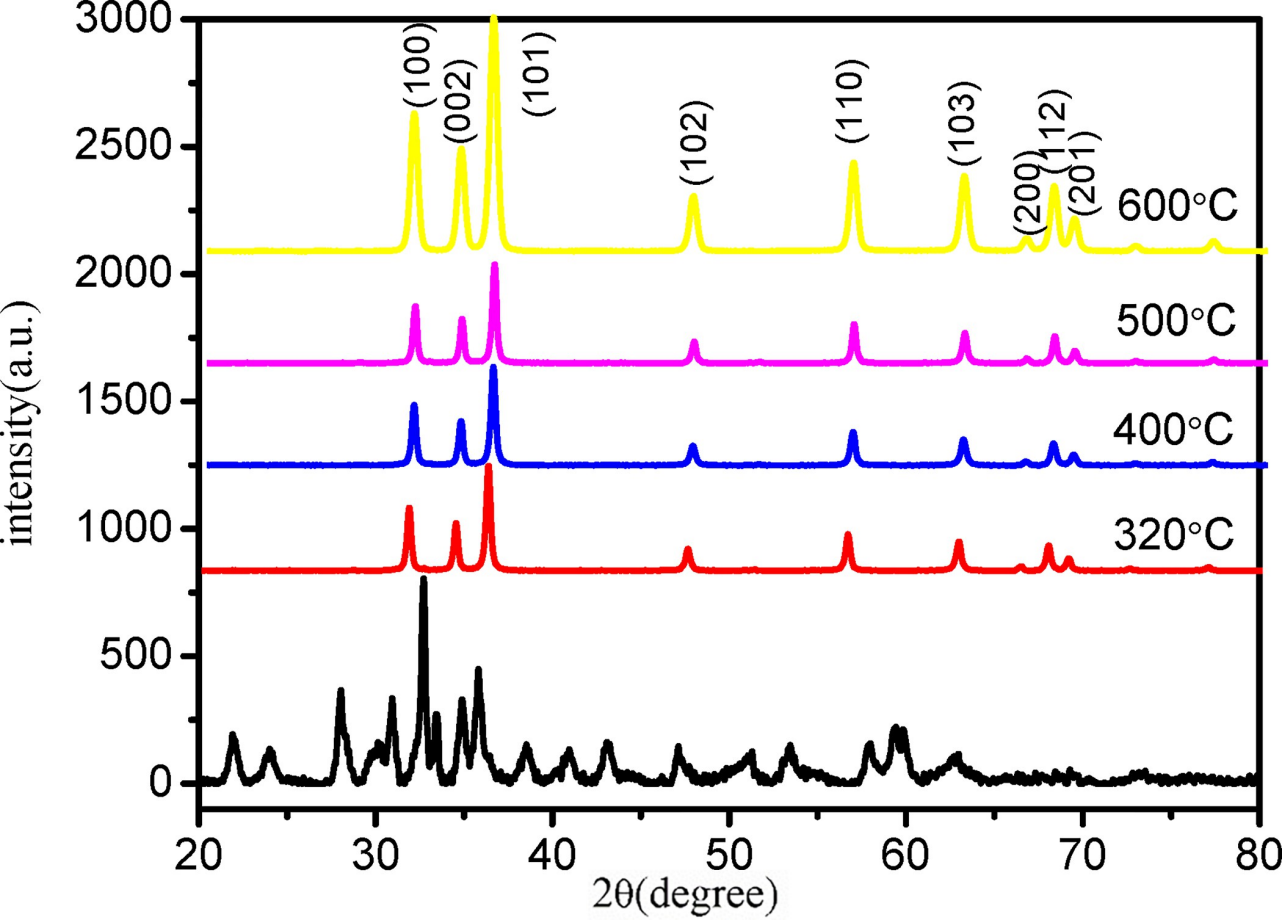
XRD of ZnO powder prepared at different calcination temperatures.

Fig 8 shows the morphology of the calcined powder. At 320°C, the ZnO powder had a spheroid-like morphology with a similar grain size of approximately 30 nm. At 400°C, the particles showed a trend of end-to-end connection and agglomeration. At 500°C, the ZnO particles formed a rod-shaped structure with end-to-end connections. At 600°C, the boundaries of the particles merged, making it difficult to distinguish the particle boundaries, and the particles were connected into a rod-like structure with a certain thickness. These results indicate that with increasing temperature, the spheroid-like ZnO powder tended to grow toward a rod-shaped polarity. Thermal calcination can cause ZnO nanoparticles to merge and form larger particles. This process is observed during the calcination of nanoparticles as zinc or oxygen defects at grain boundaries can lead to grain aggregation and merging, resulting in the formation of neck growth between adjacent particles. This is a common phenomenon [38, 43]. As the calcination temperature increases, the nucleation rate of particles also increases, resulting in a higher supersaturation of the reaction products, which accelerates the nucleation reaction in a short period of time. Consequently, the control step of the reaction shifts from grain growth to crystal nucleus formation. With the continuous increase in temperature, the phenomenon of "nuclear aggregation" caused by the rapid formation of crystal nuclei becomes more pronounced, leading to intergranular aggregation. The particle aggregation rate is the main factor that controls the morphology and crystalline structure of the final product [39].

### Effect of solvents on the properties of ZnO powder

One disadvantage of using the direct precipitation method is that it can easily lead to agglomeration of the prepared powder. To reduce the agglomeration of the powder particles, we added anhydrous ethanol to the solvent and observed the distribution and changes in particle size of the ZnO particles by varying the amount of anhydrous ethanol added.

**Fig 9:**
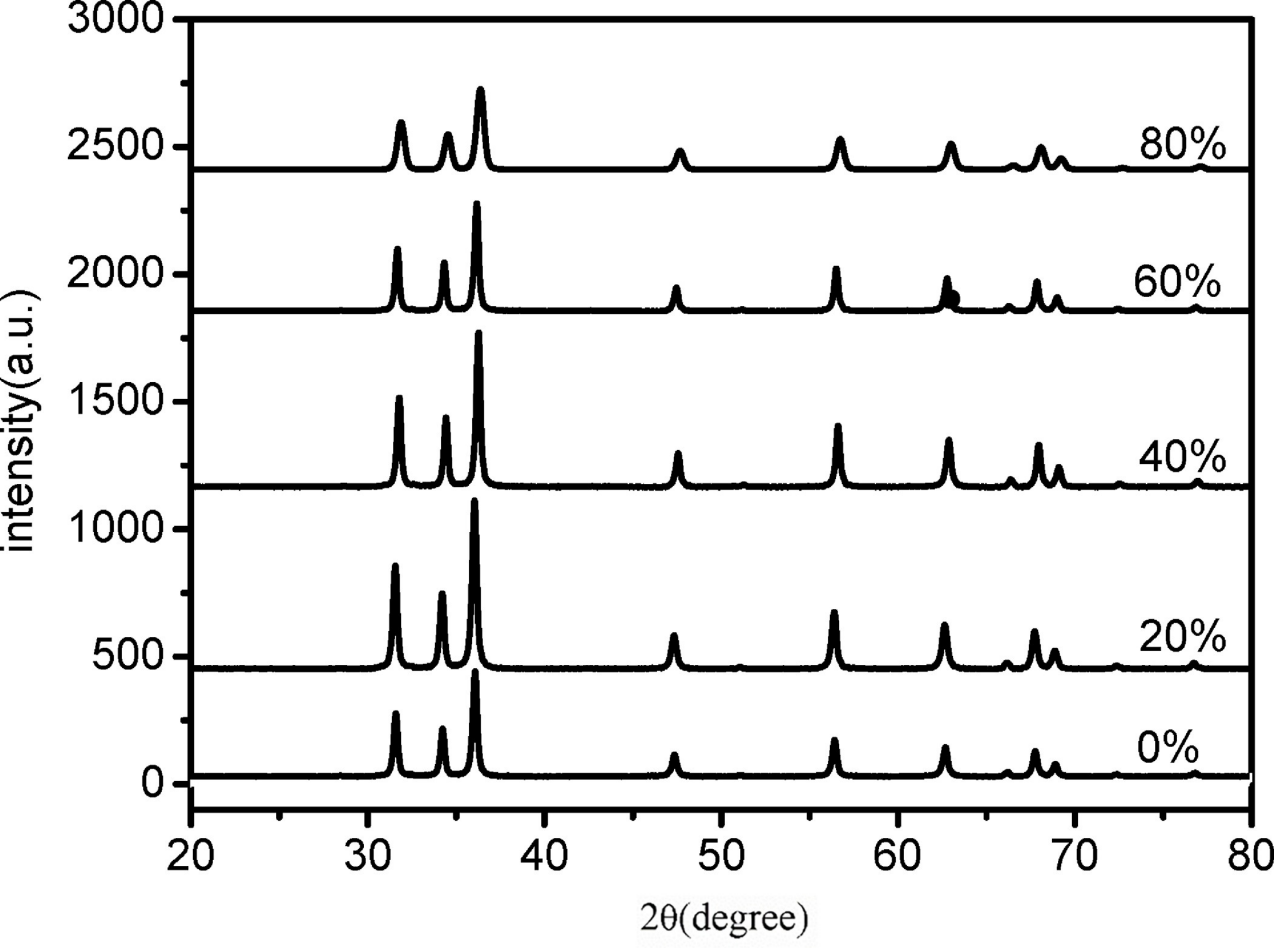
XRD of ZnO powder obtained from different anhydrous ethanol mixed solutions.

Fig 9 depicts the XRD pattern of ZnO powder prepared using anhydrous ethanol with different concentrations as a solvent. The solvents used in the experiment were ethanol-deionized water mixed solvents containing 0%, 20%, 40%, 60%, and 80% (volume ratio) deionized water, respectively. The precipitator was (NH_4_)_2_·CO_3_, and PEG-400 and TEA were used as additives. The calcination temperature and time were 320°C and 2 hours, respectively. Based on the XRD pattern in Fig 9, it can be inferred that the crystal form of ZnO powder is not affected by the ethanol concentration, but only the grain size is affected. To identify the size of nanoparticles, Scherrer’s Eq (1) was applied to peaks at 31.69°, 34.36° and 36.18° [44].


$$D=\frac{K\lambda }{\beta cos(\theta )}$$ (1)

**Fig 10:**
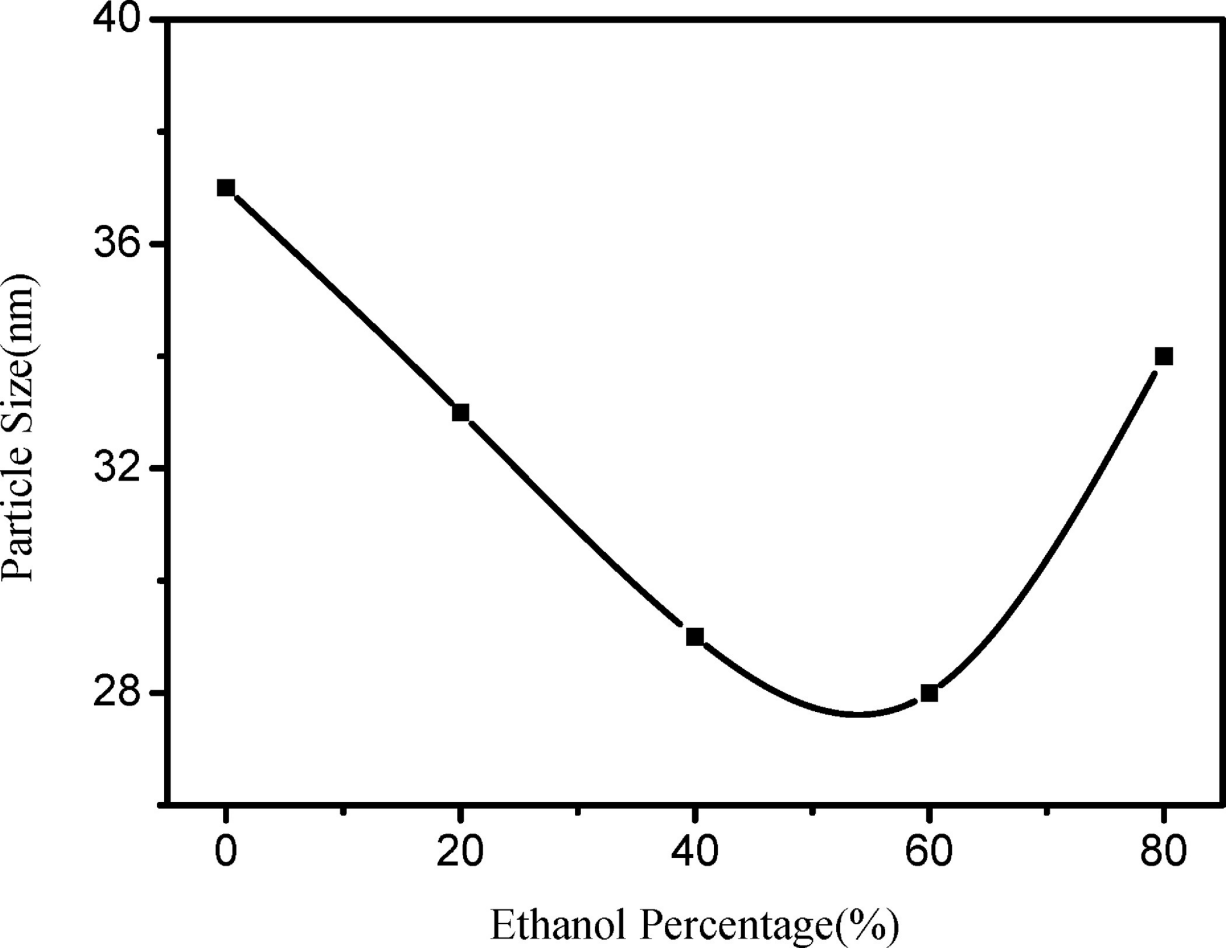
Variation trend of particle size under different ethanol ratios.

Where D is crystallite size, K is Scherrer constant and the crystallite shape factor, K = 0.9, λ is X-ray wavelength which is at 1.541 Å, β is full width at half maximum and θ is angle at maximum. The calculation demonstrated that the size of ZnO nanopowder are 37.2nm, 33.6nm, 29.3 nm, 28.1nm and 34.2nm respectively. Fig 10 illustrates the relationship between the grain size and different ethanol concentrations calculated using the Scherrer formula. As observed from Fig 10, with the increase in ethanol content, the particle size of the powder first decreases and then increases. The smallest particle size, about 28 nm, was obtained when the ethanol content was 60%. The change in microcrystalline size may be related to the change in solvent polarity, as the hydrolysis rate in solvents may vary [45].

It is widely recognized that the morphology of polar crystals can be controlled by regulating their interaction with the crystal solvent interface [46]. When highly polar solvents are used, the adsorption and deposition of precursor molecules onto the surface of polar crystals can be impeded. Since highly polar solvent molecules interact more strongly with polar crystal surfaces, the interaction between water and polar ZnO surfaces is stronger than that between ethanol and ZnO. Therefore, increasing the amount of anhydrous ethanol added to the solvent leads to a decrease in the diameter of ZnO nanorods. ZnO is formed when Zn^2+^ reacts with OH^−^. Ethanol is immiscible in aqueous solution, and its addition can separate the direct interaction between Zn^2+^ and OH^−^, thereby suppressing the nucleation of ZnO [47],When the ethanol content in the solvent is increased to 60 vol%, the average diameter of ZnO nanorods gradually decreases to 28 nm. Table 1 summarizes the published literature and shows that ZnO prepared by the ultrasonic-assisted direct precipitation method has a smaller size, a more uniform distribution, and a spherical morphology, which significantly reduces agglomeration.

**Table 1:** The effect of fabrication methods on ZnO characteristics.

Synthesis methods	Size and Shape	Precipitating Agent	Solvent	Temperature	References
Sol-Gel	Rod,81.28nm-84.98 nm	Zn(CH_3_COO)_2_·2H_2_O、NaOH	Ethanol	Not Given	[48]
Hydrothermal	Nanorods,length:2μm,diameter: 100-150nm	Zn(NO_3_)_2_·6H_2_O、C_6_H_12_N_4_	C_6_H_12_N_4_	60–95°C	[49]
Solvothermal	Cauliflower-like,15−25 nm	C_10_H_14_ZnO_4_	THF	120°C	[50]
Hydrothermal	Nanorod,150 nm	Zn(NO_3_)_2_·6H_2_O、NaOH	Ethanol	180°C	[51]
Precipitation	Spherical, 47 nm	Zn (NO_3_)_2_·6H_2_O、(NH_4_)_2_CO_3_	Water	Not Given	[52]
Microwave-ssisted hydrothermal	Needle Shape/flower-shape, 50nm–150nm	(Zn(NO_3_)_2_·6H_2_O, Zn(CH_3_COO)·2H_2_O、 N_2_H_4_、NH_3_	Water	Not Given	[53]
Electrochemical	Spherical and cylindrical, 50-200nm	Zn electrode、C_2_H_2_O_4_⋅2H_2_O、KCl, NaOH、HNO_3_.	Aqueous electrolyte	25°C	[54]
Sonochemical	Flakes shape, 200–400nm	Zn(NO_3_)_2_·6H_2_O、KOH、CTAB	Water	Room temperature	[55]
CVD	Nanorod shape, Diameter 90nm/length 564nm	Zinc acetate di-hydrate	/	650°C	[56]
Co-precipitation	Spherical, 20nm–40nm	Zn(NO_3_)_2_·4H_2_O、NH₃·H₂O	Water	Room temperature	[57]
Wet chemical	Nanodisc, 300nm-500nm	ZnCl_2_、NaOH	Water	Not Given	[58]
Ball milling	spherical, 60nm	ZnO powder	/	/	[59]

Furthermore, adding anhydrous ethanol can also improve the dispersion of the powder to some extent. Nano ZnO powders have a high specific surface area, resulting in high surface activity and strong adsorption of water molecules, which quickly react to form Zn-OH with a surface hydroxyl structure. The high surface energy and hydroxyl structure are the main reasons for the agglomeration of nano-oxide powders. Therefore, anhydrous ethanol can be used to prevent agglomeration.

## Conclusion

In this paper, the preparation of nano ZnO powder using zinc nitrate hexahydrate as a Zn source through the ultrasonic-assisted direct precipitation method was studied. The effects of precipitator type, calcination temperature, additive type and dosage, and solvent on the basic properties of ZnO powder, such as particle size, morphology, and dispersibility was full investigated. The following are the main conclusions:

1. The use of NaOH, NH_3_·H_2_O, and (NH_4_)_2_·CO_3_ as precipitants resulted in ZnO powder with a wurtzite crystal structure. The ZnO powder prepared using NaOH as a precipitant was of short rod shape with obvious agglomeration. The ZnO powder prepared using NH_3_·H_2_O as a precipitator had irregular particle shapes, large size differences, and good dispersibility. The ZnO powder prepared using (NH_4_)_2_·CO_3_ as a precipitator had circular flake-shaped particles with a size of about 50 nm and significant agglomeration.
2. Addition of different surfactants resulted in a slight shift in the position of the diffraction peak in the XRD diffraction pattern of the precursor prepared, but the phase of the precursor produced remained the same. Addition of PVP-K30 changed the particle morphology and resulted in a more uniform grain size of ZnO powder, refining its properties. Addition of 1% wt PEG-400 resulted in good powder dispersion and network distribution of particles. When the amount of PEG-400 added was 3% wt, the agglomeration was reduced, and at 5% wt, the agglomeration tendency increased. Addition of 1% wt TEA resulted in spherical particle growth with slight agglomeration and a uniform particle size distribution of about 35 nm. At 3% wt TEA, the morphology was spherical with a smooth surface, good dispersion, and a particle size distribution between 26 and 32 nm. At 5% wt TEA, the particle size of ZnO powder had little change, but the particles were connected end-to-end and tended to grow into rods.
3. The crystal forms of ZnO powders obtained at different calcination temperatures were the same. Using (NH_4_)_2_CO_3_ as a precipitator, PEG+TEA as an additive, and calcining at 320°C, we obtained ZnO powder with a spherical shape and a uniform grain size of about 30 nm. Calcining at 400°C resulted in particles with end-to-end contact, similar to agglomeration. Calcining at 500°C resulted in rod-shaped particles formed by the end-to-end connection of particles. Calcining at 600°C resulted in the fusion of the head and tail boundaries of the particles to form a rod-like structure with a certain thickness.
4. The concentration of ethanol aqueous solution did not affect the crystal form of ZnO powder, but it affected its grain size. With increasing ethanol concentration, the particle size of the powder first decreased and then increased. When the volume fraction of ethanol was 60%, the particle size was the smallest, about 28 nm.
5. Washing with anhydrous ethanol can remove a portion of the water in the precursor and occupy the gaps between the particles with anhydrous ethanol, increasing the spacing between the particles while removing water. During subsequent calcination, anhydrous ethanol can quickly volatilize, increase the particle gap, and increase the bulkiness of the particles, making the prepared ZnO powder more dispersed.

## Supporting information

S1 Raw data (RAR)
